# Prevalence, characteristics, and respiratory arousal threshold of positional obstructive sleep apnea in China: a large scale study from Shanghai Sleep Health Study cohort

**DOI:** 10.1186/s12931-022-02141-3

**Published:** 2022-09-12

**Authors:** Weijun Huang, Xiaoting Wang, Chong Xu, Huajun Xu, Huaming Zhu, Suru Liu, Jianyin Zou, Jian Guan, Hongliang Yi, Shankai Yin

**Affiliations:** 1 Department of Otorhinolaryngology Head and Neck Surgery, Shanghai Jiao Tong University Affiliated Sixth People’s Hospital, 600 Yishan Road, Shanghai, China; 2 Shanghai Key Laboratory of Sleep Disordered Breathing, 600 Yishan Road, Shanghai, China; 3 Otolaryngology Institute of Shanghai Jiao Tong University, 600 Yishan Road, Shanghai, China

**Keywords:** Positional obstructive sleep apnea, Prevalence, Respiratory arousal threshold

## Abstract

**Purpose:**

To evaluate the prevalence, characteristics, and respiratory arousal threshold (ArTH) of Chinese patients with positional obstructive sleep apnea (POSA) according to the Cartwright Classification (CC) and Amsterdam Positional Obstructive Sleep Apnea Classification (APOC).

**Methods:**

A large-scale cross-sectional study was conducted in our sleep center from 2007 to 2018 to analyze the clinical and polysomnography (PSG) data of Chinese POSA patients. Low ArTH was defined based on PSG indices.

**Results:**

Of 5,748 OSA patients, 36.80% met the CC criteria, and 42.88% the APOC criteria, for POSA. The prevalence of POSA was significantly higher in women than men (40.21% and 46.52% vs. 36.13% and 42.18% for CC and APOC, respectively). Chinese POSA patients had a lower apnea hypopnea index (AHI) and lower oxygen desaturation index, shorter duration of oxygen saturation (SaO_2_) < 90%, and a higher mean SaO_2_ and higher lowest SaO_2_ value compared to subjects with non-positional OSA (NPOSA). More than 40% of the POSA patients had a low ArTH; the proportion was extremely high in the supine-isolated-POSA (si-POSA) group and APOC I group. In multivariate logistic regression analyses, higher mean SaO_2_ and lower AHI during sleep were positive predictors of POSA.

**Conclusions:**

According to the CC and APOC criteria, more than 1/3 of our Chinese subjects with OSA had POSA. Chinese POSA patients had less severe OSA and nocturnal hypoxia. Compared to NPOSA patients, significantly more patients with POSA had a low ArTH. A low ArTH may be an important endotype in the pathogenesis of POSA, especially in patients with si-POSA and APOC I. Further studies are necessary to develop personalized management strategies for POSA patients.

*Trial registration:* Chinese Clinical Trial Registry; URL: http://www.chictr.org.cn; No. ChiCTR1900025714 (retrospectively registered).

**Supplementary Information:**

The online version contains supplementary material available at 10.1186/s12931-022-02141-3.

## Introduction

Studies have shown that as many as 9–38% of adults suffer from long-term sleep disorders [1, 2]. Obstructive sleep apnea (OSA) is a common sleep disorder with serious adverse health consequences [3–5]. There are currently about 176 million OSA patients in China, with about 66 million classified as moderate to severe [1]. OSA can be classified as positional OSA (POSA) or non-positional OSA (NPOSA) according to whether the occurrence of respiratory events is associated with the body position during sleep. The Cartwright Classification (CC) is the most commonly used POSA standard [6], while the Amsterdam Positional Obstructive Sleep Apnea Classification (APOC) is a newer set of classification criteria [7].

The results regarding the prevalence of POSA vary between studies with different criteria and cohorts. According to the CC criteria, Asian studies showed that 28–80% of OSA in adults is POSA [8–14], and studies in Western countries showed that the ratio exceeded 50% not only in adults [7, 8, 15–20], but also in children [21] and the elderly [22]. Anatomical and non-anatomical factors contribute differently to OSA between Chinese and Caucasian patients [23, 24]. Therefore, the prevalence of POSA in ethnic Chinese populations is likely to differ from that in other races. However, there has been only one previous study of Chinese POSA patients, which used single criterion, had a small sample size and limited subgroup analyses, and did not adjust for confounding factors [14]. Thus, the prevalence of POSA in the general Chinese population, and the characteristics of those with the condition, remain unknown. Due to the increasing socioeconomic burden of OSA in China, it is essential to identify the clinical characteristics of Chinese POSA patients to provide individualized treatment.

It is important to elucidate the pathophysiology of POSA to develop optimal treatment methods [25]. A low respiratory arousal threshold (ArTH), i.e., easy arousal from sleep in response to relatively mild airway obstruction, is one of the non-anatomical physiological factors associated with OSA [26–28]. Patients with a low ArTH are unlikely to be adherent to continuous positive airway pressure (CPAP) treatment [29]. Therefore, it is important to understand the prevalence and effects of non-anatomical traits in OSA. However, data regarding the pathogenesis of POSA, especially with ArTH, in large clinical populations remain scarce. This large-scale study was performed to evaluate the prevalence, clinical characteristics, and ArTH of POSA patients in China, and to identify possible predictors of POSA.

## Methods

### Subject recruitment

Study subjects were enrolled between May 15, 2007, and December 31, 2018, at our sleep center as part of the Shanghai Sleep Health Study cohort. This study was conducted in accordance with the Declaration of Helsinki and the study protocol was approved by the Ethics Committee of Shanghai Jiao Tong University Affiliated Sixth People’s Hospital (Approval No: 2019-KY-050[K]). The study was registered at the Chinese Clinical Trial Registry (No. ChiCTR1900025714). All subjects provided informed consent.

Ethnic Chinese participants aged ≥ 18 years with snoring or daytime sleepiness and undergoing laboratory polysomnography (PSG), were screened for eligibility for inclusion in the study. OSA subjects who had been treated previously or had other comorbid sleep disorders (insomnia, narcolepsy, upper airway resistance syndrome, or restless legs syndrome) were excluded. Subjects taking anxiolytics, antidepressants, antipsychotics, or hypnotic drugs were also excluded. Data from participants with total sleep time (TST) ≥ 4 h, sleep time in each position ≥ 30 min, and ≥ 10% of the TST in both the best sleeping position (BSP) and worst sleeping position (WSP) were considered suitable for inclusion in the study. All subjects completed a comprehensive questionnaire pertaining to alcohol consumption, smoking, and medication use, before PSG.

### Clinical evaluation

Height (m), weight (kg), neck circumference (NC) (cm), waist circumference (WC) (cm), hip circumference (HC) (cm), and blood pressure (mmHg) were recorded as the mean values of two consecutive measurements before PSG. Body mass index (BMI) was calculated as weight divided by height squared (kg/m^2^). Fasting blood samples were taken from each subject the morning after PSG. The glycolipid metabolism index was measured in our laboratory. The homeostasis model assessment of insulin resistance (HOMA-IR) index was calculated as fasting insulin (μU/mL) multiplied by fasting glucose (mmol/L) and the result was divided by 22.5 [30]. Subjects diagnosed by their physician and using antihypertensive or antiarrhythmic medications were considered to have hypertension or cardiovascular disease (CVD). The diagnoses of diabetes and hyperlipidemia relied on past history and the lipid index, according to the 2016 ESC/EAS Guidelines for the Management of Dyslipidemias [31]. Metabolic syndrome (MS) was defined according to International Diabetes Federation guidelines [32]. Participants completed the Epworth Sleepiness Scale (ESS). Those with an ESS score > 10 were considered to have excessive daytime sleepiness (EDS) [33].

### Sleep evaluation and POSA classification

An Alice 4, 5, or 6 Sleep Diagnostic System (Respironics Inc., Pittsburgh, PA, USA) was used for nocturnal monitoring for full-night laboratory PSG. During the laboratory-based PSG, electroencephalogram, electrooculogram, electrocardiogram, and electromyogram recordings were obtained. Nasal airflow was measured using a nasal pressure cannula, and blood oxygen saturation was measured by a finger pulse oximeter. A belt containing a piezoelectric transducer was used to record chest and abdominal movements. An accelerator-based position sensor placed at the sternum was used to distinguish among the supine, prone, right, left, and upright positions with simultaneous infrared video recording (reviewed only in ambiguous cases) [34]. The video recording was not performed only if the subject refused to provide permission due to the reason of privacy protection. All data were recorded automatically and continuously from 22:00 to 06:00. Two experienced technicians checked the data and output reports manually using Sleepware software (Respironics Inc.) according to the American Academy of Sleep Medicine (AASM) 2007 guidelines [35]. Patients with an apnea hypopnea index (AHI) ≥ 5 events/h were included in the OSA group, while subjects with AHI < 5 events/h were considered non-OSA subjects. OSA was classified as mild (AHI ≥ 5, < 15/h), moderate (AHI ≥ 15, < 30/h), severe (AHI ≥ 30, < 55/h), or extremely severe (AHI ≥ 55/h) [36]. Data on AHI in the supine position, AHI in non-supine positions, the microarousal index (MAI), TST, sleep efficacy, proportion of each sleep stage, such as the rapid eye movement (REM) stage and non-rapid eye movement (NREM) stage (consisting of N1, N2, slow wave sleep [SWS], and N3), the time in each position during sleep, oxygen desaturation index (ODI), cumulative time of oxygen saturation < 90% in TST (CT90), mean oxygen saturation (SaO_2_), and lowest oxygen saturation (LSaO_2_) were also collected.

The CC criteria for POSA include a supine AHI at least double that of the non-supine position, and a sleep time in each position ≥ 30 min. If these criteria are not met, OSA is classified as NPOSA. [6] We further classified POSA as supine-isolated POSA (si-POSA; AHI ≥ 5/h, supine AHI ≥ 2 non-supine AHI, and non-supine AHI < 5/h) or supine-predominant POSA (sp-POSA; AHI ≥ 5/h, supine AHI ≥ 2 non-supine AHI, and non-supine AHI ≥ 5/h) [24].

The APOC criteria require a sleep time ≥ 10% of the TST in both the BSP and WSP (APOC I: AHI of BSP < 5/h; APOC II: AHI of BSP in inferior OSA severity compared to overall AHI; APOC III: AHI of BSP at least 25% lower than the overall AHI and overall AHI ≥ 40/h) [7, 37].

### ArTH

Edwards et al. established a clinical screening tool for low ArTH based on three criteria: AHI < 30/h, LSaO_2_ > 82.5%, and proportion of hypopneas > 58.3% [38]. Each fulfilled criterion is scored as 1, and a total score ≥ 2 is taken to indicate a low ArTH. This tool is widely used and does not require epiglottic measurements, thus allowing analyses of large retrospective datasets [39–41]. The equation for determining the ArTH is as follows (where male sex = 1 and female sex = 0:

$$ArTH=-65.39+\left(0.06*age\right)+\left(3.69*sex\right)-\left(0.03*BMI\right)-\left(0.11*AHI\right)+\left(0.53*LSaO2\right)+ (0.09*proportion of hypopneas)$$

### Statistical analysis

The sample size was determined based on power analysis. With a power of 90% and α of 0.05, 2,300 participants were required. The Kolmogorov–Smirnov test was used to verify the normality of the data distribution. Continuous variables with a normal distribution are shown as means ± standard deviation, while skewed data are presented as the median (first to third quartile). Categorical data are presented as percentages. The data were further analyzed by ANOVA, *t* test, Kruskal–Wallis test, and the χ^2^ test. Logistic regression analyses were performed to identify predictors of POSA, and the association between ArTH and POSA. Age, BMI, sex, NC, WC, HC, alcohol consumption, smoking, TST, ESS, MAI, and CT90 were included as potential confounding factors. Statistical analyses were performed using SPSS software (version 25.0; IBM Corp., Armonk, NY, USA). In all analyses, *P* < 0.05 was taken to indicate statistical significance.

## Results

### Prevalence of POSA

**Fig. 1:**
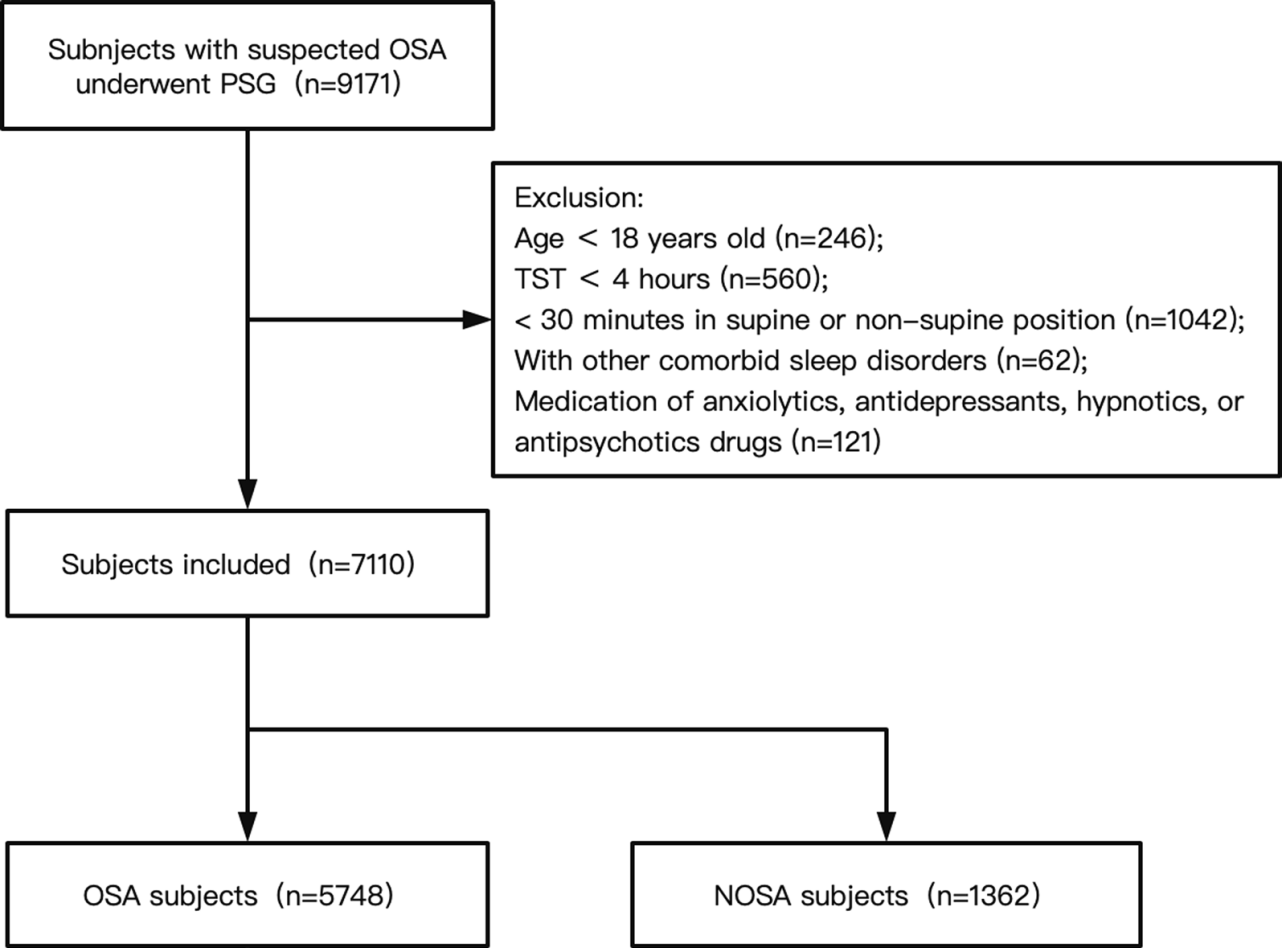
Flow diagram of the recruitment process. *NOSA* non-obstructive sleep apnea, *OSA* obstructive sleep apnea, *PSG* polysomnography, *TST* total sleep time

**Fig. 2:**
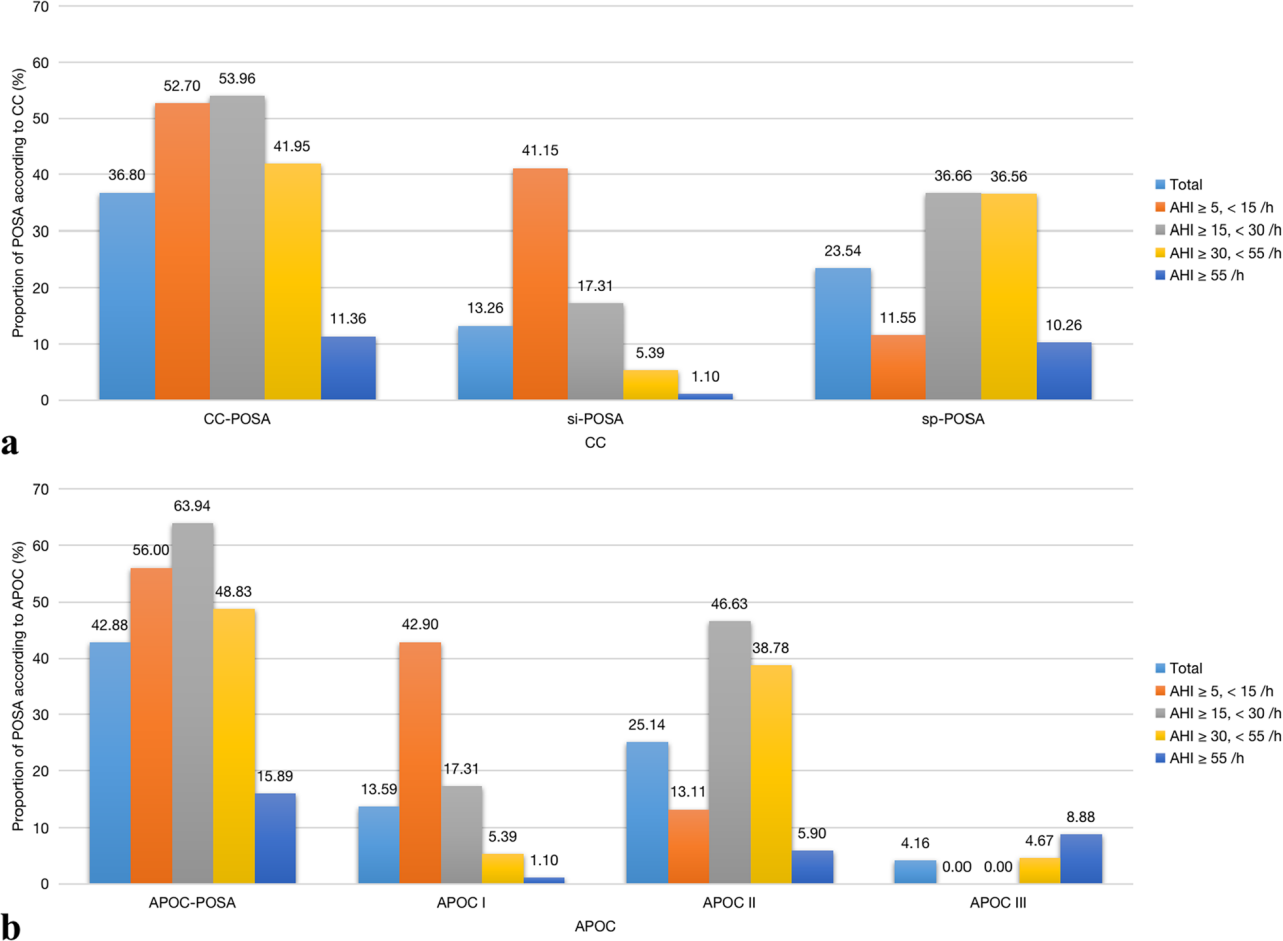
Prevalence of CC-POSA **a** and APOC-POSA **b** in different OSA severity groups. *AHI* apnea–hypopnea index, *APOC* Amsterdam Positional Obstructive Sleep Apnea Classification, *CC* Cartwright Classification, *POSA* positional obstructive sleep apnea, *si-POSA* supine-isolated positional obstructive sleep apnea, *sp-POSA* supine-predominant positional obstructive sleep apnea

**Fig. 3:**
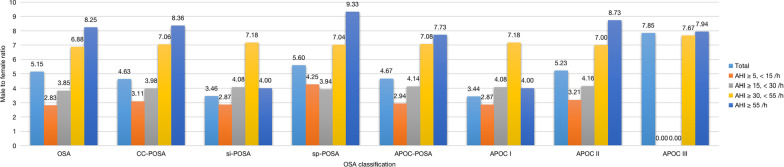
Male to female ratio of OSA and POSA subjects in different OSA severity groups. *AHI* apnea–hypopnea index, *APOC* Amsterdam Positional Obstructive Sleep Apnea Classification; *CC* Cartwright Classification, *OSA* obstructive sleep apnea, *POSA* positional obstructive sleep apnea, *si-POSA* supine-isolated positional obstructive sleep apnea, *sp-POSA* supine-predominant positional obstructive sleep apnea

**Fig. 4:**
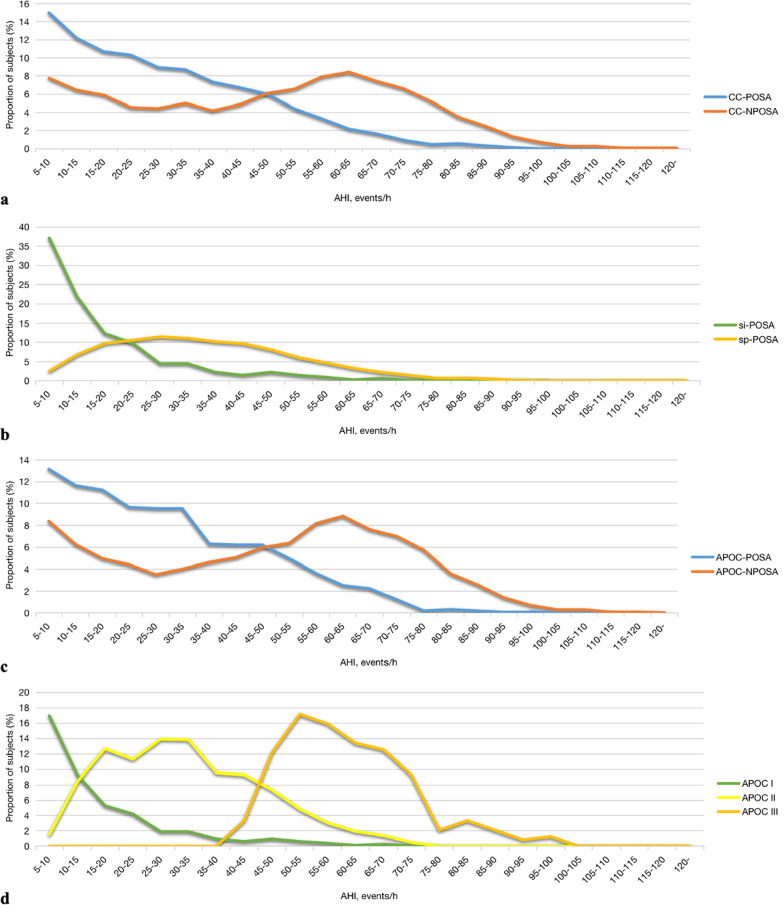
Treads in the proportions of POSA subjects with AHI **a** CC-POSA and CC-NPOSA, **b** Si-POSA and sp-POSA, **c** APOC-POSA and APOC-NPOSA, **d** APOC I, APOC II and APOC III. *AHI* apnea–hypopnea index, *APOC* Amsterdam Positional Obstructive Sleep Apnea Classification, *CC* Cartwright Classification, *NPOSA* non-positional obstructive sleep apnea, *POSA* positional obstructive sleep apnea, *si-POSA* supine-isolated positional obstructive sleep apnea, *sp-POSA* supine-predominant positional obstructive sleep apnea

Of the 9,171 patients in our study cohort with suspected OSA between May 15, 2007, and December 31, 2018, 2,061 were excluded due to inappropriate age (< 18 years, *n* = 246), TST < 4 h (*n* = 560), < 30 min spent in the supine or non-supine position (*n* = 1,042), the presence of comorbid sleep disorders (*n* = 62), and taking anxiolytics, antidepressants, hypnotics, or antipsychotic drugs (*n* = 121). Therefore, the final study sample consisted of 7,110 patients (Fig. 1) of whom the clinical and sleep characteristics were showed in Additional file 2: Table S1. Of the 5,748 OSA patients, 36.80% met the CC criteria, and 42.88% the APOC criteria, for POSA (Fig. 2). The prevalence of POSA was significantly higher in women than men according to both the CC (Table 1) and APOC (Table 2) criteria (40.21% and 46.52% in women vs. 36.13%, 42.18% in men, respectively). According to the CC and APOC criteria, the male to female ratios were 8.36:1 and 7.73:1 for extremely severe POSA, and 3.11:1 and 2.94:1 for mild OSA, respectively (Fig. 3). The male to female ratio of POSA increased with increasing AHI. The prevalence of POSA decreased significantly with increasing AHI (Fig. 4a, c) with similar trends according to both the CC and APOC criteria. The prevalence of si-POSA was higher in mild OSA than sp-POSA (Fig. 4b). APOC I was associated with milder AHI than APOC II and III (Fig. 4d). The APOC grade increased with AHI and nocturnal hypoxia severity.

**Table 1:** Clinical and Sleep Characteristics of OSA Subjects (n = 5748) according to CC

Characteristics	CC-POSA (n = 2115)	Si-POSA (n = 762)	Sp-POSA (n = 1353)	CC-NPOSA (n = 3633)
Demographic and clinical characteristics				
Men, n (%)	1739 (82.22)	591 (77.56)	1148 (84.85)^###^	3074 (84.61)*
In man, n (%)	1739/4813 (36.13)	591/4813 (12.28)	1148/4813 (23.85)^###^	3074/4813 (63.87)***
In women, n (%)	376/935 (40.21)	171/935 (18.29)	205/935 (21.92)^##^	559/935 (59.79)***
Age, yrs	44 (36–54)	43 (35–54)	45 (36–54)	43 (35–53)**
BMI, kg/m2	26.47 (24.51–28.73)	25.71 (23.72–27.72)	26.98 (25.06–29.29)^###^	27.51 (25.15–30.03)***
NC, cm	40 (37–41)	38.50 (36–41)	40 (38–42)^###^	40 (38–42.50)***
WC, cm	95 (90–101)	92.5 (88–98)	97 (91–103)^###^	98 (92–105)***
HC, cm	100 (96–105)	99 (95–103)	101 (97–106)^###^	103 (98–108)***
WHR	0.94 (0.90–0.98)	0.94 (0.90–0.97)	0.95 (0.92–0.99)^###^	0.96 (0.91–0.99)***
SBP, mmHg	120 (118–132)	120 (120–125)	123 (120–135)^###^	123 (120–136)***
DBP, mmHg	80 (77–85)	80 (79–80)	80 (76–85)^#^	80 (78–89)***
Hypertension, n (%)	549 (25.96)	166 (21.78)	383 (28.31)^#^	1021 (28.10)*
Diabetes mellitus, n (%)	185 (8.75)	59 (7.74)	126 (9.31)	300 (8.26)
CVD, n (%)	150 (7.09)	46 (6.04)	104 (7.69)	301 (8.29)
MS, n (%)	372 (17.59)	110 (14.44)	262 (19.36)^#^	645 (17.75)***
Hyperlipidemia, n (%)	369 (17.45)	106 (13.91)	263 (19.44)	631 (17.37)
Smoking, n (%)	470 (22.22)	165 (21.65)	305 (22.54)	685 (18.11)**
Alcohol consumption, n (%)	951 (44.96)	337 (44.23)	614 (45.38)	2077 (57.17)***
Snoring score, point	6 (5–8)	6 (4–8)	7 (5–9)^###^	7 (5–9)***
ESS, point	7 (3–11)	7 (3–11)	7 (3–12)	9 (4–14)***
EDS, n (%)	615 (29.08)	198 (25.98)	418 (30.89)	1418 (39.03)***
Biochemical indicators				
Fasting glucose, mmol/L	5.25 (4.89–5.74)	5.16 (4.86–5.60)	5.29 (4.91–5.81)^###^	5.35 (4.97–5.95)***
Glucose 120 min, mmol/L	8.54 (6.28–11.84)	8 (6.02–11.84)	8.58 (6.48–11.90)	7.78 (6.29–11.47)
Fasting insulin, µU/mL	10.74 (7.23–15.47)	9.61 (6.54–13.61)	11.33 (7.83–16.65)^###^	12.68 (8.43–18.57)***
Insulin 120 min, µU/mL	64.15 (39.35–119.50)	60.80 (34.72–134.23)	66.97 (40.52–115.75)	71.49 (45.42–131)*
HOMA-IR	2.35 (1.49–3.65)	2.14 (1.36–3.21)	2.53 (1.58–4.03)^###^	2.88 (1.72–4.54)***
TC, mmol/L	4.71 (4.16–5.33)	4.64 (4.12–5.28)	4.75 (4.19–5.36)^#^	4.81 (4.22–5.44)**
TG, mmol/L	1.63 (1.15–2.33)	1.49 (1.06–2.07)	1.71 (1.20–2.45)^###^	1.74 (1.22–2.52)***
HDL, mmol/L	1.02 (0.90–1.18)	1.06 (0.90–1.23)	1.01 (0.90–1.15)^###^	1 (0.87–1.15)***
LDL, mmol/L	2.94 (2.45–3.46)	2.92 (2.40–3.40)	2.96 (2.48–3.50)	2.98 (2.47–3.53)
ApoA-1, g/L	1.05 (0.94–1.18)	1.07 (0.95–1.19)	1.04 (0.94–1.17)	1.05 (0.95–1.18)
ApoB, g/L	0.84 (0.73–0.97)	0.82 (0.71–0.95)	0.85 (0.74–0.98)^###^	0.87 (0.75–1)***
ApoE, mg/dL	4.27 (3.46–5.26)	4.10 (3.37–5.12)	4.35 (3.51–5.38)^##^	4.40 (3.58–5.58)***
Lp (α), mg/dL	7.70 (3.90–16.10)	8.60 (4.20–18.70)	7.20 (3.80–14.70)^##^	7.20 (3.80–15.35)
ApoA/ApoB	1.26 (1.05–1.51)	1.30 (1.09–1.58)	1.24 (1.04–1.48)^###^	1.22 (1.03–1.46)***
PSG				
Mild OSA, n (%)	575 (27.19)	449 (58.92)	126 (9.31^)###^	516 (14.21)***
Moderate OSA, n (%)	633 (29.93)	203 (26.64)	430 (31.78)^###^	540 (14.86)***
Severe OSA, n (%)	701 (33.14)	90 (11.81)	611 (45.16)^###^	970 (26.70)***
Extreme severe OSA, n (%)	206 (9.74)	20 (2.62)	186 (13.75)^###^	1607 (44.23)***
AHI, events/h	25.80 (14.30–41.30)	12.55 (7.90–22.03)	34.30 (22.55–46.20)^###^	50.60 (25.30–66.75)***
OAHI, events/h	11.35 (4.75–22.37)	5.22 (2.33–10.74)	16.19 (8.35–26.89)^###^	26.07 (10.32–44.20)***
Longest time of obstructive respiratory event, second	45 (32.50–59)	36.50 (25.50–51)	49.50 (37–62.50)^###^	57 (42–71)***
AHIREM, events/h	31.10 (12.20–51.40)	16 (5.60–33.70)	40 (21.40–56.40)^###^	51.40 (30–64.50)***
AHINREM, events/h	25.20 (12.95–42.40)	12.30 (7.03–22.68)	33.60 (20.70–47.05)^###^	49.80 (23–67.20)***
Supine AHI, events/h	42.1 (24.40–60.30)	21.40 (12.48–35.55)	53.20 (38.10–65.70)^###^	41.40 (5.90–67.50)***
Non-supine AHI, events/h	7.80 (3.20–16.70)	2.26 (1.17–3.46)	13.42 (8.30–21.88)^###^	46.35 (24.57–64.04)***
ODI, events/h	26.20 (13.70–41.80)	13 (8–23.25)	33.90 (22.30–48.10)^###^	49.80 (23.80–68.45)***
Mean SaO2, %	95 (94–96)	96 (94–96)	94 (93–95)^###^	94 (91–95)***
LSaO2, %	82 (75–87)	86 (81–89)	80 (72–84)^###^	74.50 (64–82)***
CT90, % TST	2.84 (0.76–7.97)	0.92 (0.23–3.42)	4.48 (1.54–10.31)^###^	9.61 (2.23–27.53)***
TST, min	411.50 (347.48–452.50)	410 (347–453)	413.50 (347.45–452.50)	425 (372–470.50)***
Supine Time, % TST	73.39 (46.57–84.56)	72 (49.79–83.75)	73.77 (44.17–85.05)	75.89 (38.43–85.85)
Sleep efficiency, %	94.52 (87.07–98.64)	94.47 (86.50–98.85)	94.59 (87.50–98.50)	96.12 (88.81–99.33)***
N1, % TST	16 (8.50–24.50)	14.50 (7.30–23.63)	16.40 (8.80–25.55)^##^	15.40 (7.10–25.90)
N2, % TST	50.90 (38.10–59.40)	50.70 (38.48–59.10)	51 (37.75–59.65)	51.30 (37.70–61.50)
SWS, % TST	12.50 (5.60–19.50)	13.30 (6.38–20.40)	11.90 (5.30–19.20)^#^	10.80 (3.40–20.10)**
REM, % TST	10.40 (5.90–15)	10.80 (6.07–15.20)	10.30 (5.65–14.90)	9.80 (5.70–14.20)*
MAI, events/h	21.70 (12.60–34.60)	18.20 (11.10–28.30)	24.40 (14–37.90)^###^	27.30 (14.90–49.40)***
Components of ArTH score				
AHI < 30 events/h, n (%)	1208 (57.12)	652 (85.56)	556 (41.09)^###^	1056 (29.07)***
LSaO2 > 82.5%, n (%)	989 (46.76)	503 (66.01)	486 (35.92)^###^	891 (24.53)***
Proportion of hypopneas > 58.3%, n (%)	660 (31.21)	301 (39.50)	359 (26.53)^###^	740 (20.37)***
Absolute fraction of hypopnoeas, %	40 (17.31–64.77)	47.17 (22.24–73.72)	35.85 (15.48–60.25)^###^	27.02 (8.20–51.98)***
ArTH Score, cmH2O	− 16.44 (− 21.62− [-11.78])	− 12.43 (− 16.80− [− 8.49])	− 18.53 (− 23.58− [− 14.18])^###^	-23.66 (-30.84-[-16.17])***
Proportion with ArTH score ≥ 2, n (%)	973 (46)	542 (71.13)	431 (31.86)^###^	830 (22.85)***
In male, n (%)	750/1739 (43.13)	410/591 (69.37)	340/1148 (29.62)^###^	616/3074 (20.04)***
In female, n (%)	223/376 (59.31)	132/171 (77.19)	91/205 (44.39)^###^	214/559 (38.28)***

*OSA* obstructive sleep apnea, *CC* Cartwright Classification, *CC-POSA* positional obstructive sleep apnea according to Cartwright Classification; *Si-POSA* supine-isolated positional obstructive sleep apnea, *Sp-POSA* supine-predominant positional obstructive sleep apnea, *CC-NPOSA* non-positional obstructive sleep apnea according to Cartwright Classification; *BMI* body mass index; *NC* neck circumference, *WC* waist circumference; *HC* hip circumference; *WHR* waist hip ratio; *SBP* systolic blood pressure; *DBP* diastolic blood pressure; *CVD* cardiovascular diseases, *MS* metabolic syndrome; ESS, Epworth Sleepiness Scale; EDS, excessive daytime sleepiness; HOMA-IR, homeostasis model assessment of insulin resistance, *TC*, total cholesterol; TG, triglyceride; HDL, high-density lipoprotein cholesterol; *LDL* low-density lipoprotein cholesterol; *ApoA-I* apolipoprotein A-I; *ApoB* apolipoprotein B; *ApoE* apolipoprotein E; Lp (a), lipoprotein (a); *PSG* polysomnography, *AHI* apnea hypopnea index, *OAHI* obstructive apnea hypopnea index; AHIREM, apnea hypopnea index in rapid eye movement stage; AHINREM, apnea hypopnea index in non-rapid eye movement stage; ODI, oxygen desaturation index; SaO2, oxygen saturation; LSaO2, lowest oxygen saturation; CT90, the cumulative time spent at oxygen saturation below 90% in total sleep time; TST, total sleep time; SWS, slow wave sleep; REM, rapid eye movement; MAI, micro-arousal index; ArTH, respiratory arousal threshold. *Indicated p-value < 0.05 between POSA and NPOSA in subgroup analysis. **Indicated p-value < 0.05 between POSA and NPOSA in subgroup analysis. ***Indicated p-value < 0.001 between POSA and NPOSA in subgroup analysis.^#^Indicated p-value < 0.05 between si-POSA and sp-POSA in subgroup analysis. ^##^Indicated p-value < 0.05 between si-POSA and sp-POSA in subgroup analysis. ^###^Indicated p-value < 0.001 between si-POSA and sp-POSA in subgroup analysis

**Table 2:** Clinical and Sleep Characteristics of OSA Subjects (n = 5748) according to APOC

﻿Characteristics	APOC-POSA (n = 2465)	APOC I (n = 781)	APOC II (n = 1445)	APOC III (n = 239)	APOC-NPOSA (n = 3283)
Demographic and clinical characteristics					
Men, n (%)	2030 (82.35)	605 (77.46)	1213 (83.94)	212 (88.70)^###^	2783 (84.77)*
In man (%)	2030/4813 (42.18)	605 (12.57)	1213 (25.20)	212 (4.41)	2783 (57.82)***
In women (%)	435/935 (46.52)	176 (12.82)	232 (24.81)	27 (2.89)	500 (53.48)**
Age, yrs	44 (35–54)	43 (35–54)	45 (36–55)	42 (35–51)^#^	43 (35–53)*
BMI, kg/m2	26.49 (24.56–28.73)	25.71 (23.72–27.71)	26.73 (24.86–29.05)	28.15 (26.18–30.46)^###^	27.64 (25.17–30.07)***
NC, cm	40 (37.50–41.50)	38.50 (36–41)	40 (38–42)	41 (39–43)^###^	40 (38–42.50)***
WC, cm	96 (90–101)	92 (88–98)	96 (91–102)	100 (96–107)^###^	99 (93–105)***
HC, cm	101 (97–105)	99 (95–103)	101 (97–106)	104 (100–109)^###^	103 (98–108)***
WHR	0.94 (0.90–0.98)	0.93 (0.89–0.97)	0.95 (0.91–0.98)	0.97 (0.93–1)^###^	0.96 (0.92–0.99)***
SBP, mmHg	120 (119–132)	120 (119–125)	122 (120–134)	126 (120–138)^###^	123 (120–136)***
DBP, mmHg	80 (76–85)	80 (79–81)	80 (75–85)	80 (76–88)^###^	80 (78–89)***
Hypertension, n (%)	630 (25.56)	168 (21.51)	401 (27.75)	61 (25.52)^##^	940 (28.63)**
Diabetes mellitus, n (%)	215 (8.72)	60 (7.68)	136 (9.41)	19 (7.95)	270 (8.22)
CVD, n (%)	176 (7.14)	47 (6.02)	118 (8.17)	11 (4.60)	275 (8.38)
MS, n (%)	444 (18.01)	111 (14.21)	260 (17.99)	73 (30.54)^###^	573 (17.45)***
Hyperlipidemia, n (%)	441 (17.89)	107 (13.70)	262 (18.13)	72 (30.13)^###^	559 (17.03)
Smoking, n (%)	547 (22.19)	170 (21.77)	304 (21.04)	73 (30.54)^##^	608 (18.52)***
Alcohol consumption, n (%)	1113 (45.15)	348 (44.56)	668 (46.23)	97 (40.59)	1915 (58.33)***
Snoring score, point	6 (5–8)	6 (4–8)	6 (5–8)	8 (5–9)^###^	7 (5–9)***
ESS, point	7 (3–12)	7 (3–11)	7 (2–12)	9 (4–13)^###^	9 (4–14)***
EDS, n (%)	726 (29.45)	198 (25.35)	431 (29.83)	97 (40.59)^###^	1363 (41.52)***
Biochemical indicators					
Fasting glucose, mmol/L	5.25 (4.89–5.73)	5.16 (4.85–5.59)	5.27 (4.90–5.74)	5.43 (4.93–6.21)^###^	5.37 (4.98–5.96)***
Glucose 120 min, mmol/L	8.25 (6.24–11.73)	8.09 (6.07–11.82)	8.35 (6.28–11.73)	7.19 (6.32–10.58)	7.97 (6.35–11.50)
Fasting insulin, µU/mL	10.90 (7.45–15.79)	9.63 (6.56–13.70)	11.11 (7.74–16.51)	13 (9.95–20.19)^###^	12.85 (8.46–18.65)***
Insulin 120 min, µU/mL	67.86 (41.43–120.40)	63 (35.57–138.90)	66.79 (41.15–118.95)	81.46 (63.62–123.78)	71.01 (44.07–127.90)
HOMA-IR	2.38 (1.52–3.72)	2.13 (1.33–3.21)	2.45 (1.56–3.90)	3.03 (2.08–5.02)^###^	2.93 (1.72–4.56)***
TC, mmol/L	4.72 (4.16–5.32)	4.64 (4.11–5.28)	4.76 (4.20–5.36)	4.67 (4.08–5.29)^#^	4.82 (4.22–5.47)***
TG, mmol/L	1.65 (1.15–2.33)	1.49 (1.06–2.08)	1.70 (1.19–2.42)	1.84 (1.27–2.55)^###^	1.74 (1.23–2.54)***
HDL, mmol/L	1.02 (0.90–1.18)	1.06 (0.90–1.23)	1.02 (0.90–1.16)	0.98 (0.86–1.10)^###^	1 (0.87–1.15)***
LDL, mmol/L	2.94 (2.44–3.47)	2.91 (2.40–3.40)	2.97 (2.48–3.50)	2.91 (2.43–3.52)	2.99 (2.47–3.53)*
ApoA-1, g/L	1.05 (0.94–1.18)	1.07 (0.95–1.19)	1.04 (0.94–1.18)	1.03 (0.93–1.15)^#^	1.05 (0.95–1.18)
ApoB, g/L	0.84 (0.73–0.97)	0.82 (0.71–0.95)	0.85 (0.74–0.98)	0.81 (0.73–0.99)^##^	0.87 (0.76–1)***
ApoE, mg/dL	4.28 (3.47–5.30)	4.09 (3.39–5.12)	4.32 (3.52–5.34)	4.49 (3.60–5.85)^###^	4.41 (3.59–5.60)***
Lp (α), mg/dL	7.85 (3.90–16.30)	8.60 (4.16–18.74)	7.60 (3.90–15.10)	6.80 (3.48–15.75)^#^	7.12 (3.80–14.90)*
ApoA/ApoB	1.26 (1.06–1.50)	1.30 (1.09–1.58)	1.25 (1.04–1.48)	1.23 (1.06–1.47)^###^	1.21 (1.02–1.46)***
PSG					
Mild OSA, n (%)	611 (24.79)	468 (59.92)	143 (9.90)	0 (0)^###^	480 (14.62)***
Moderate OSA, n (%)	750 (30.43)	203 (25.99)	547 (37.85)	0 (0)^###^	423 (12.89)***
Severe OSA, n (%)	816 (33.10)	90 (11.53)	648 (44.85)	78 (32.64)^###^	855 (26.04)***
Extreme severe OSA, n (%)	288 (11.68)	20 (2.56)	107 (7.40)	161 (67.36)^###^	1525 (46.45)***
AHI, events/h	27.30 (15–43.20)	12.20 (7.70–21.70)	30.70 (20.60–41.80)	61.10 (52.20–70.10)^###^	52.10 (26–67.60)***
OAHI, events/h	12.03 (5.19–23.70)	4.95 (2.23–10.58)	14.35 (7.76–24.01)	34.51 (23.81–47.85)^###^	27.27 (10.75–44.93)***
Longest time of obstructive respiratory event, second	46 (33.50–60)	36.50 (25.50–51)	49 (36–62)	57.50 (47–71.50)^###^	57.50 (42.50–71.48)***
AHIREM, events/h	33 (13.20–52.30)	15.40 (5.60–33.60)	37.10 (20.20–53.10)	58.45 (46.18–70.80)^###^	52.40 (30.90–65.10)***
AHINREM, events/h	26.25 (13.90–43.43)	12 (6.85–22.60)	30.10 (19.39–42.70)	59.65 (49.90–70.93)^###^	51.55 (23.95–68.12)***
Supine AHI, events/h	41.20 (24.10–59.65)	20.90 (11.85–34.80)	46.30 (32.80–60.30)	71.30 (62.10–80.80)^###^	43.70 (2–68.60)***
Non-supine AHI, events/h	9.70 (3.76–20.57)	2.30 (1.20–3.54)	13.42 (8.78–20.93)	36.36 (32.69–42.77)^###^	49.59 (27.13–65.70)***
ODI, events/h	27.30 (14.80–43.60)	12.70 (7.70–22.80)	30.70 (20.80–43.40)	60.60 (48.70–72.65)^###^	51.90 (25–69.50)***
Mean SaO2, %	95 (93–96)	96 (94–96)	95 (93–96)	93 (91–94)^###^	93 (91–95)***
LSaO2, %	82 (74–86)	86 (81–89)	81 (74–85)	72 (64–78)^###^	74 (64–82)***
CT90, % TST	3.09 (0.83–8.57)	0.90 (0.23–3.42)	3.87 (1.32–8.85)	13.49 (6.31–27.75)^###^	10.72 (2.49–28.95)***
TST, min	413.20 (352.10–453.50)	410 (347.75–453)	415.30 (352.43–452.50)	417.50 (359–456.50)	426 (372.50–471.90)***
Supine Time, % TST	74.04 (47.91–84.90)	72.35 (49.76–83.66)	73.70 (44.92–85.16)	80.06 (58.61–87.20)^###^	75.53 (36.01–85.79)**
Sleep efficiency, %	94.46 (86.73–98.68)	94.48 (86.52–98.86)	94.61 (87.09–98.63)	94.08 (84.56–98.43)	96.26 (89.53–99.39)***
N1, % TST	16.20 (8.50–25.20)	14.60 (7.30–23.55)	16.70 (8.80–26.30)	16.40 (9.10–26.80)^##^	15.20 (6.90–25.70)*
N2, % TST	50.80 (37.50–59.40)	50.70 (38.60–59.05)	50.80 (37.10–59.55)	51.90 (35.40–60.20)	51.30 (38.10–61.70)**
SWS, % TST	12.40 (5.50–19.60)	13.20 (6.45–20.05)	11.90 (5.20–19.40)	10.40 (3.80–18.40)^#^	10.80 (3.30–20.10)**
REM, % TST	10.30 (5.90–14.90)	10.80 (6.05–15.20)	10.20 (5.75–14.90)	9.80 (6–14.30)	9.80 (5.70–14.20)*
MAI, events/h	22.20 (12.70–35.30)	18.25 (11.13–28.28)	23.40 (13.50–36.05)	33.90 (18.80–52.80)^###^	27.80 (15–50.20)***
Components of ArTH score					
AHI < 30 events/h, n (%)	1361 (55.21)	671 (85.92)	690 (47.75)	0 (0)	903 (27.51)
LSaO2 > 82.5%, n (%)	1108 (44.95)	518 (66.33)	560 (38.75)	30 (12.55)	772 (23.52)
Proportion of hypopneas > 58.3%, n (%)	760 (30.83)	313 (40.08)	408 (28.24)	39 (16.32)	640 (19.49)
Absolute fraction of hypopnoeas, %	38.99 (16.67–63.95)	47.83 (21.90–73.61)	37.13 (16.04–61.51)	27.40 (8.82–49.44)	26.23 (7.66–50.75)
ArTH Score, cmH2O	− 16.69 (− 22.29-[-11.97])	− 12.32 (− 16.74− [− 8.44])	− 17.85 (− 22.65− [− 13.37])	− 25.73 (− 30.98− [− 21.97])	− 24.15 (− 31.19− [− 16.50])
Proportion with ArTH score ≥ 2, n (%)	1099 (44.58)	559 (71.57)	534 (36.96)	6 (2.51)	704 (21.44)
In male, n (%)	845/2030 (41.63)	422/605 (69.75)	418/1213 (34.46)	5/212 (2.36)	521/2783 (18.72)
In female, n (%)	254/435 (58.39)	137/176 (77.84)	116/232 (50)	1/27 (3.70)	183/500 (36.60)

*OSA* obstructive sleep apnea, *APOC* Amsterdam Positional Obstructive Sleep Apnea Classification, *APOC-POSA* positional obstructive sleep apnea according to Amsterdam Positional Obstructive Sleep Apnea Classification, *APOC-NPOSA* non-positional obstructive sleep apnea according to Amsterdam Positional Obstructive Sleep Apnea Classification, *BMI* body mass index, *NC* neck circumference; *WC* waist circumference, *HC* hip circumference, *WHR* waist hip ratio, *SBP* systolic blood pressure, *DBP* diastolic blood pressure, *CVD* cardiovascular diseases, *MS* metabolic syndrome; *ESS* Epworth Sleepiness Scale, *EDS* excessive daytime sleepiness, *HOMA-IR* homeostasis model assessment of insulin resistance, *TC* total cholesterol; TG, triglyceride, *HDL* high-density lipoprotein cholesterol, *LDL* low-density lipoprotein cholesterol, *ApoA-I* apolipoprotein A-I, *ApoB* apolipoprotein B; ApoE, apolipoprotein E; Lp (a), lipoprotein (a); *PSG* polysomnography, *AHI* apnea hypopnea index, *OAHI* obstructive apnea hypopnea index; AHIREM, apnea hypopnea index in rapid eye movement stage; AHINREM, apnea hypopnea index in non-rapid eye movement stage; ODI, oxygen desaturation index; SaO2, oxygen saturation; LSaO2, lowest oxygen saturation; CT90, the cumulative time spent at oxygen saturation below 90% in total sleep time; *TST* total sleep time, *SWS* slow wave sleep, *REM* rapid eye movement, *MAI* micro-arousal index; ArTH, respiratory arousal threshold. *Indicated p-value < 0.05 between POSA and NPOSA in subgroup analysis. **Indicated p-value < 0.05 between POSA and NPOSA in subgroup analysis. *** indicated p-value < 0.001 between POSA and NPOSA in subgroup analysis.^#^Indicated p-value < 0.05 among the three groups of APOC I, II, and III in subgroup analysis. ^##^Indicated p-value < 0.05 among the three groups of APOC I, II, and III in subgroup analysis. ^###^Indicated p-value < 0.001 among the three groups of APOC I, II, and III in subgroup analysis

### Comparison between POSA and NPOSA according to the CC and APOC criteria

**Fig. 5:**
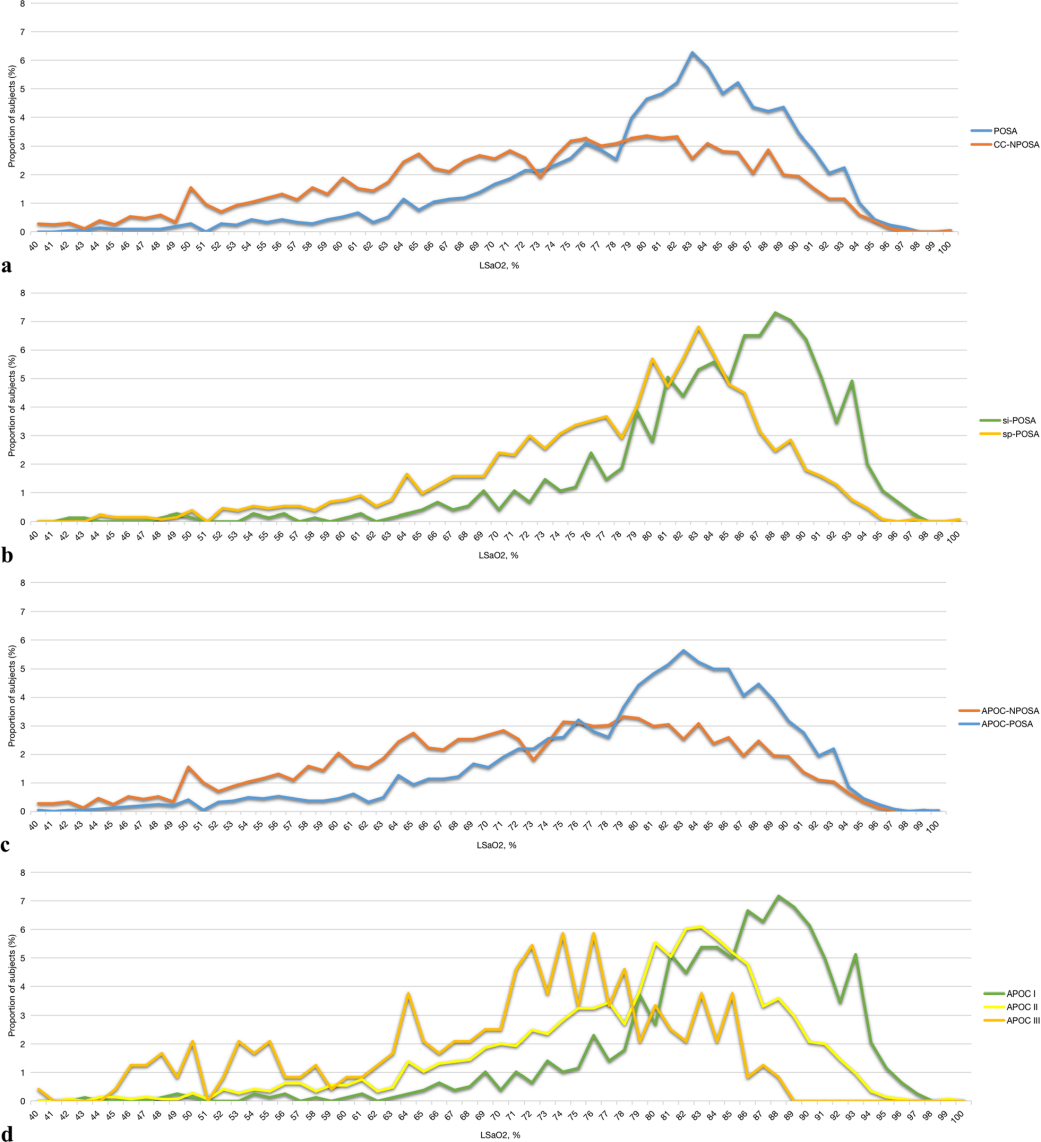
Treads in the proportions of POSA subjects with LSaO2 **a** CC-POSA and CC-NPOSA, **b** Si-POSA and sp-POSA, **c** APOC-POSA and APOC-NPOSA, **d** APOC I, APOC II and APOC III. *APOC* Amsterdam Positional Obstructive Sleep Apnea Classification, *CC* Cartwright Classification, *LSaO2* lowest oxygen saturation, *NPOSA* non-positional obstructive sleep apnea, *POSA* positional obstructive sleep apnea, *si-POSA* supine-isolated positional obstructive sleep apnea, *sp-POSA* supine-predominant positional obstructive sleep apnea

Chinese POSA patients had lower AHI, ODI, and CT90 values, and higher mean SaO_2_ and LSaO_2_ values, than NPOSA patients during sleep. The overall AHI, supine AHI, non-supine AHI, REM AHI, and NREM AHI values were lower in the POSA group (all *P* < 0.001), indicating less severe OSA compared to the NPOSA group according to both the CC (Table 1) and APOC (Table 2) criteria. Compared to the NPOSA group, the ODI and CT90 values were lower, and the mean SaO_2_ and LSaO_2_ were higher, in the POSA group, indicating less severe nocturnal hypoxia in the latter group according to both the CC (Table 1, Fig. 5a, b) and APOC (Table 2, Fig. 5c, d) criteria.

In further subgroup analyses, the si-POSA group had lower overall AHI, REM AHI, NREM AHI, supine AHI, non-supine AHI, ODI, and CT90 values, and higher mean SaO_2_ and LSaO_2_ values, than the sp-POSA group (Table 1). In subgroup analyses of APOC, the APOC I group had the lowest overall AHI, REM AHI, NREM AHI, and AHI values in the supine and non-supine positions (Table 2). ODI and CT90 were lowest, and the mean SaO_2_ and LSaO_2_ were highest, in the APOC I group, indicating that nocturnal hypoxia was less severe than in the other APOC subgroups (Table 2).

Subjects with POSA were less likely to feel sleepy and experience EDS, while the si-POSA (Table 1) and APOC I (Table 2) groups had low proportions of EDS. The POSA group had a lower BMI than the NPOSA group (26.47kg/m^2^ [24.51–28.73]kg/m^2^ vs. 27.51kg/m^2^ [25.15–30.03]kg/m^2^ and 26.49kg/m^2^ [24.56–28.73]kg/m^2^ vs. 27.64kg/m^2^ [25.17–30.07]kg/m^2^ for CC [Table 1] and APOC [Table 2], respectively). As shown in Table 1, the si-POSA group had a lower BMI than the sp-POSA group, and the BMI was lower in the APOC I group than the other APOC subgroups (Table 2). As BMI increased, the prevalence of POSA decreased (Additional file 1: Fig. S1), especially in the si-POSA and APOC I groups.

With regard to sleep structure, the percentages of SWS and REM stage sleep were significantly higher in the POSA than NPOSA group according to the CC (Table 1) and APOC (Table 2) criteria (*P* <  0.01 for SWS, *P <* 0.05 for REM stage sleep [for both criteria sets]).  Furthermore, the si-POSA group had a higher percentage of SWS and lower percentage of N1 stage sleep than the sp-POSA group. Meanwhile, the percentage of SWS was significantly higher, and the percentage of N1 stage sleep was significantly lower, in the APOC I group than APOC II and APOC III groups. The POSA group had a shorter TST and lower sleep efficiency according to the CC (Table 1) and APOC (Table 2) criteria (all *P* < 0.001). The APOC III group had a significantly higher percentage of supine sleep than the APOC I and APOC II groups (Table 2).

### ArTH

A low ArTH was more common in females than males (Table 1 and Table 2). The POSA group had a higher absolute fraction of hypopneas. The percentages of AHI < 30 events/h, LSaO_2_ > 82.5%, and hypopnea proportion > 58.3%, were higher in the POSA than NPOSA group, and higher in the si-POSA than sp-POSA group. In the si-POSA and sp-POSA groups, the proportions of low ArTH (71.13% and 31.86%, respectively) were significantly higher compared to the NPOSA group (22.85%) (Table 1). The APOC I group had the highest proportion of low ArTH (71.57%) among the three APOC subgroups (Table 2).

The results of binary logistic regression analysis of the association between POSA and low ArTH are shown in Table 3. In the model adjusted for age, BMI, and sex, si-POSA (adjusted odds ratio [OR], 7.302; 95% confidence interval [CI], 6.090–8.757; P < 0.001) and sp-POSA (adjusted OR, 1.567; 95% CI, 1.357–1.809) were significantly associated with the development of low ArTH. In a model adjusted for more potential confounders, si-POSA (adjusted OR, 3.542; 95% CI, 2.862–4.384; P < 0.001) was still significantly associated with the development of low ArTH, although significance disappeared for sp-POSA. With regard to APOC (Table 3), APOC I (adjusted OR, 3.900; 95% CI, 3.141–4.842) and II (adjusted OR, 1.287; 95% CI, 1.091–1.518) were associated with a higher likelihood of a low ArTH after adjustment for confounding factors, while APOC III was not (adjusted OR, 0.143; 95% CI, 0.062–0.330).

**Table 3:** Adjusted ORs and 95% CIs for the Association between POSA and ArTH according to CC and APOC

Predictors	n	OR (95% CI)		
		Model 1	Model 2	Model 3
CC				
CC-NPOSA	3633	Reference	Reference	Reference
Si-POSA	762	7.302 (6.090–8.757)***	6.990 (5.791–8.437)***	3.542 (2.862–4.384)***
Sp-POSA	1353	1.567 (1.357–1.809)***	1.546 (1.331–1.795)**	1.043 (0.882–1.233)
APOC				
APOC-NPOSA	3283	Reference	Reference	Reference
APOC I	781	8.159 (6.791–9.802)***	7.828 (6.471–9.471)***	3.900 (3.141–4.842)***
APOC II	1455	2.134 (1.853–2.457)***	2.098 (1.812–2.429)***	1.287 (1.091–1.518)**
APOC III	239	0.104 (0.046–0.236)***	0.118 (0.052–0.268)***	0.143 (0.062–0.330)***

Model 1 was adjusted for age, BMI, and sex. Model 2 was adjusted for variables included in Model 1 and NC, WC, HC, alcohol consumption, smoking. Model 3 was adjusted for variables included in Model 2 and TST, ESS, MAI, CT90. *OR* odds ratio, *CI* confidence interval, *POSA* positional obstructive sleep apnea; ArTH, respiratory arousal threshold; CC, Cartwright Classification; *APOC* Amsterdam Positional Obstructive Sleep Apnea Classification; *CC-NPOSA* non-positional obstructive sleep apnea according to Cartwright Classification, *Si-POSA* supine-isolated positional obstructive sleep apnea; Sp-POSA, supine-predominant positional obstructive sleep apnea; APOC-NPOSA, non-positional obstructive sleep apnea according to Amsterdam Positional Obstructive Sleep Apnea Classification, *BMI* body mass index; *NC* neck circumference, *WC* waist circumference; HC, hip circumference; TST, total sleep time, *ESS* Epworth Sleepiness Scale, *MAI* micro-arousal index, *CT90* the cumulative time spent at oxygen saturation below 90% in total sleep time. ** indicated p-value < 0.05 and *** indicated p-value < 0.001 for the logistic regression. NPOSA group was the reference category in each subgroup analysis

### POSA predictors

Univariate and multivariate regression analyses demonstrated that a higher mean SaO_2_ (adjusted OR, 1.099; 95% CI, 1.063–1.136 for CC; and adjusted OR, 1.086; 95% CI, 1.053–1.120 for APOC) and lower AHI (adjusted OR, 0.967; 95% CI, 0.960–0.973 for CC; and adjusted OR, 0.968; 95% CI, 0.961–0.974 for APOC) were positive predictors of POSA (Table 4). Table 5 shows the results of binary logistic regression analysis of the associations among mean SaO_2_, AHI, and POSA. Compared to AHI ≥ 55/h, mild OSA (5 ≤ AHI < 15/h), moderate OSA (15 ≤ AHI < 30/h) and 30 ≤ AHI < 55/h were more likely to be associated with POSA according to the CC (adjusted OR, 4.174; 95% CI, 3.238–5.380; adjusted OR, 4.818; 95% CI, 3.826–6.066; and adjusted OR, 3.643; 95% CI, 2.977–4.457, respectively). Similar results were found for APOC (adjusted OR, 3.400; 95% CI, 2.673–4.325; adjusted OR, 5.127; 95% CI, 4.121–6.379; and adjusted OR, 3.399; 95% CI, 2.822–4.095, respectively) (Table 5). After adjusting for potential confounders, OSA patients with a mean SaO_2_ > 95% were 49.5% (OR, 1.495 [95% CI, 1.075–2.080]) and 44.3% (OR, 1.443 [95% CI, 1.053–1.976]) more likely to have POSA according to CC or APOC, respectively, than those with a mean SaO_2_ < 92% (Table 5).

**Table 4:** Adjusted ORs and 95% CIs for the Association Between Predictors and POSA according to CC and APOC

Predictors (n = 5748)	OR (95% CI) of CC		OR (95% CI) of APOC	
	Univariate regression analyses	Multivariate regression analyses	Univariate regression analyses	Multivariate regression analyses
Age	1.006 (1.002–1.011)*		1.006 (1.005–1.007)*	
Women	1.189 (1.030–1.372)*		1.181 (1.151–1.211)***	
BMI	0.936 (0.923–0.950)***		0.918 (0.916–0.920)***	
NC	0.929 (0.915–0.944)***		0.918 (0.915–0.920)***	
WC	0.971 (0.966–0.977)***		0.964 (0.964–0.965)***	
HC	0.969 (0.962–0.976)***		0.964 (0.963–0.966)***	
ESS	0.963 (0.954–0.972)***		0.954 (0.952–0.955)***	
AHI	0.965 (0.962–0.968)***	0.967 (0.960–0.973)***	0.957 (0.956–0.957)***	0.968 (0.961–0.974)***
ODI	0.969 (0.967–0.972)***		0.964 (0.964–0.965)***	
Mean SaO2	1.269 (1.239–1.299)***	1.099 (1.063–1.136)***	1.259 (1.254–1.263)***	1.086 (1.053–1.120)***
LSaO2	1.055 (1.050–1.061)***		1.060 (1.059–1.061)***	

*OR* odds ratio, *CI* confidence interval, *POSA* positional obstructive sleep apnea, *CC* Cartwright Classification, *APOC* Amsterdam Positional Obstructive Sleep Apnea Classification, *BMI* body mass index; *NC* neck circumference, *WC* waist circumference, *HC* hip circumference, *ESS* Epworth Sleepiness Scale; AHI, apnea hypopnea index; ODI, oxygen desaturation index, *SaO2* oxygen saturation, *LSaO2* lowest oxygen saturation. * indicated p-value < 0.05, ** indicated p-value < 0.05 and *** indicated p-value < 0.001 for the logistic regression

**Table 5:** Adjusted ORs and 95% CIs for the Association Between AHI / Mean SaO2 and POSA according to CC and APOC

Predictors	n	OR (95% CI)		
		Model 1	Model 2	Model 3
CC-AHI				
5 ≤ AHI < 15/h	1091	8.630 (7.075–10.526)***	8.445 (6.859–10.397)***	4.174 (3.238–5.380)***
15 ≤ AHI < 30/h	1173	9.056 (7.485–10.957)***	9.093 (7.460–11.083)***	4.818 (3.826–6.066)***
30 ≤ AHI < 55/h	1671	5.574 (4.665–6.661)***	5.634 (4.685–6.775)***	3.643 (2.977–4.457)***
AHI ≥ 55 /h	1813	Reference	Reference	Reference
Mean SaO2				
Mean SaO2 > 95%	2412	5.795 (4.781–7.024)***	6.837 (5.569–8.394)***	1.495 (1.075–2.080)*
94 ≤ Mean SaO2 ≤ 95	984	4.252 (3.435–5.264)***	4.787 (3.826–5.989)***	1.324 (0.965–1.816)
92 ≤ Mean SaO2 < 94	1130	2.923 (2.370–3.606)***	3.165 (2.542–3.941)***	1.236 (0.939–1.627)
Mean SaO2 < 92	1222	Reference	Reference	Reference
APOC-AHI				
5 ≤ AHI < 15/h	1091	6.606 (5.490–7.948)***	6.582 (5.415–7.999)***	3.400 (2.673–4.325)***
15 ≤ AHI < 30/h	1173	9.175 (7.673–10.973)***	9.489 (7.869–11.443)***	5.127 (4.121–6.379)***
30 ≤ AHI < 55/h	1671	4.997 (4.250–5.875)***	5.161 (4.359–6.110)***	3.399 (2.822–4.095)***
AHI ≥ 55 /h	1813	Reference	Reference	Reference
Mean SaO2				
Mean SaO2 > 95%	2412	5.256 (4.406–6.270)***	6.371 (5.268–7.706)***	1.443 (1.053–1.976)*
94 ≤ Mean SaO2 ≤ 95	984	4.108 (3.370–5.008)***	4.700 (3.812–5.795)***	1.337 (0.989–1.807)
92 ≤ Mean SaO2 < 94	1130	2.685 (2.215–3.256)***	2.960 (2.418–3.625)***	1.178 (0.910–1.527)
Mean SaO2 < 92	1222	Reference	Reference	Reference

Model 1 was adjusted for age, BMI, and sex. Model 2 was adjusted for variables included in Model 1 and NC, WC, HC, alcohol consumption, smoking. Model 3 was adjusted for variables included in Model 2 and TST, ESS, MAI, CT90. *OR* odds ratio, *CI* confidence interval, *AHI* apnea hypopnea index, *SaO2* oxygen saturation, *POSA* positional obstructive sleep apnea, *CC* Cartwright Classification, *APOC* Amsterdam Positional Obstructive Sleep Apnea Classification; BMI, body mass index; *NC* neck circumference, *WC* waist circumference, *HC* hip circumference, *TST* total sleep time, *ESS* Epworth Sleepiness Scale; MAI, micro-arousal index; CT90, the cumulative time spent at oxygen saturation below 90% in total sleep time. *Indicated p-value < 0.05, **Indicated p-value < 0.05 and ***Indicated p-value < 0.001 for the logistic regression. Group of AHI ≥ 55 /h and group of Mean SaO2 < 92% were the reference categories in subgroup analysis, respectively

## Discussion

To our knowledge, this is the first study to analyze the clinical characteristics and ArTH of Chinese POSA patients according to both the CC and APOC criteria, and the prevalence of the disease in a large Chinese sample. More than 1/3 of the OSA subjects met the CC or APOC criteria for POSA, representing a lower prevalence than in Western studies. In addition, more than 40% of the POSA patients had a low ArTH. The proportion was extremely high in the si-POSA and APOC I groups.

Compared to NPOSA, POSA patients are less obese and have less severe OSA [8, 10, 13, 24]. Certain craniofacial characteristics, such as retrognathia, have been shown to promote upper airway obstruction in Chinese patients regardless of BMI. Cephalometric studies reported a shorter cranial base, maxilla, and mandible in Chinese OSA patients [42], along with a more posteriorly positioned mandible and inferiorly positioned hyoid bone, and an enlarged tongue and soft palate, compared to Caucasian OSA patients [43]. One study found that the maximum esophageal pressure was significantly higher in Asians than Caucasians [44]. These findings have been attributed to the greater craniofacial restriction seen in Asians [42]. A smaller upper airway is more likely to collapse, thereby promoting OSA in both the supine and non-supine positions [45].

In POSA, respiratory events generally cease, accompanied by cortical arousal. Respiratory arousal during sleep can prevent apnea and may even be lifesaving [41, 46]. However, low ArTH prevents deeper sleep stages (SWS) with stable breathing, and leads to ventilatory instability with sleep fragmentation even under conditions of mild upper airway obstruction [28, 47, 48]. Premature arousal results in inadequate chemical stimuli for activation of the upper airway dilator muscles [28]. Therefore, a low ArTH is an important endotype in the pathogenesis of POSA [27].

Non-obese OSA patients, who have a high prevalence of low ArTH, also tend to have less collapsible upper airways and less severe OSA. The characteristics of these patients are identical to those of POSA patients [49]. Strategies to increase the ArTH have the potential to make breathing more stable during sleep. Previous studies showed that increasing the ArTH through pharmacological interventions can reduce OSA severity, particularly in patients with a low initial ArTH [50]. Compared to Caucasians, Asians are less likely to exhibit a low ArTH [41]. The findings suggest that genetic background is likely a key factor underlying pathophysiological traits that are predisposing factors for OSA [51]. This is supported by emerging evidence that compromised upper airway anatomy in Asians is predominantly due to a restriction caused by the craniofacial skeletal structure, whereas in African-Americans it is primarily due to enlargement of upper airway soft tissues in the setting of obesity as well as non-anatomical factors [52].

A low ArTH may be significant factor in terms of the pathogenesis of OSA in nonobese patients, and is a strong predictor of poor compliance with long-term CPAP use. This emphasizes the importance of understanding the pathophysiological phenotype of OSA for treatment management and enhanced CPAP compliance [49]. In one study, at least one-third of OSA patients had low ArTH levels [53]. In the present study, over 40% of the POSA patients had low ArTH levels. POSA is generally less severe, and this finding may explain why POSA patients have poorer compliance with CPAP treatment [49, 54].

Our patients with a higher mean SaO_2_ (> 95%) during sleep and mild-to-moderate OSA were more likely to have POSA. Women with OSA had a higher likelihood of POSA, especially those in the si-POSA and APOC I groups, suggesting that women may benefit more from positional therapy. Although positional therapy alone will not resolve upper airway obstruction in the majority of OSA patients, it could be combined with treatments that improve ArTH and loop gain as an alternative to CPAP in certain POSA patients [55]. Eszopiclone, zopiclone, and zolpidem were found to increase ArTH in previous randomized controlled trials [25, 56]. Therefore, these medications may improve compliance with CPAP therapy in POSA patients.

The present study had a number of strengths, including analysis of most of the relevant clinical and PSG characteristics of POSA patients, and the performance of full-night PSG in the laboratory. Moreover, the CC and APOC criteria were both applied, with adjustment for potential confounding factors to avoid false-negative results. Finally, the large sample size allowed full subgroup analyses.

However, this study also had some limitations. First, it could only demonstrate an association between POSA and ArTH due to its observational design. As epiglottic pressure measurement (the gold standard ArTH evaluation) is extremely difficult in a large-scale study, a validated clinical screening tool was used for subgroup analyses, and to determine subtle clinical associations. Although this screening tool is widely accepted, it was developed in a largely non-ethnic Chinese population and the potential for bias effect should be acknowledged. The equation proposed for calculating ArTH may not be applicable to other populations, as the role of ArTH in the pathogenesis and treatment of OSA may vary by morphology, age, and ethnicity [41]. As the major determinants of OSA severity in Chinese patients are anatomical, rather than non-anatomical, the ArTH may have been slightly overestimated for a given level of OSA severity which is similar in non-ethnic Chinese population. Our indirect estimations of ArTH should be verified via invasive measurements directly in ethnic Chinese subjects. Also, POSA was diagnosed based on recordings performed for only 1 night, where considerable night-to-night variability in respiratory events has been reported; OSA severity (according to ODI) changed in 77.9% of patients [57]. And 19.7% of subjects were misdiagnosed when using an ODI cutoff of 15 events/h during single-night PSG [58]. The intraindividual variability (indicated by the coefficient of variation) was > 30%, allowing for the identification of a relevant number of OSA patients who would have been misdiagnosed or misclassified with single-night sleep study [57, 58]. As OSA severity exhibits a considerable night-to-night variability, the sleep position that determines the phenotype of POSA might show similar variability. Recording the sleeping position for several consecutive nights may be necessary to confirm the POSA phenotype. Nonetheless, this study adds to the literature by shedding light on the prevalence of POSA in China, and the clinical characteristics and ArTH of Chinese POSA patients.

## Conclusion

Among the subjects with OSA included in this study, 36.80% and 42.88% met the CC and APOC criteria for POSA, respectively. Chinese POSA patients had less severe OSA and nocturnal hypoxia compared to the si-POSA and APOC I groups. In comparison with NPOSA patients, significantly more patients with POSA had a low ArTH. A low ArTH may be an important endotype in the pathogenesis of POSA. Further studies are necessary to develop personalized management strategies for patients with POSA.

## Current knowledge/study rationale

There is strong evidence that the severity of obstructive sleep apnea (OSA) can worsen when sleeping in the supine position, which is known as positional OSA (POSA). While POSA is prevalent among adults, there are limited data on the presence and characteristics of POSA in China. In addition, studies of large clinical populations examining how the respiratory arousal threshold (ArTH), a key physiological trait, is involved in the pathogenesis of POSA, which may influence adherence to continuous positive airway pressure (CPAP), are lacking. This study was performed to assess the prevalence, characteristics, and ArTH of POSA in a large Chinese cohort.

## Study impact

More than 1/3 of our Chinese subjects with OSA had POSA, which was especially prevalent among those with mild OSA. The rate was lower than in Western studies because of the differences in anatomical and non-anatomical factors between Chinese and Western populations. A low ArTH is more common in patients with POSA compared to those with non-positional OSA (NPOSA). These data suggest that millions of Chinese people have POSA, where assessment of ArTH can help identify patients at risk of poor CPAP adherence, and may inform the selection of targeted therapy to improve CPAP use. Personalized treatment, such as the use of positional therapy devices, should be considered when treating POSA, and may be beneficial for individuals with poor adherence to CPAP.

## Supplementary Information

**Additional file 1: Figure S1.** Prevalence of CC-POSA (a) and APOC-POSA (b) by BMI. APOC, Amsterdam Positional Obstructive Sleep Apnea Classification; BMI, body mass index; CC, Cartwright Classification; POSA, positional obstructive sleep apnea; si-POSA, supine-isolated positional obstructive sleep apnea; sp-POSA, supine-predominant positional obstructive sleep apnea.

**Additional file 2: Table S1.** Clinical and Sleep Characteristics of All Subjects (n = 7110).

## Data Availability

The datasets used and analyzed in this study are available from Hongliang Yi, the corresponding author, on reasonable request.

## Abbreviations

AASM: American Academy of Sleep Medicine
AHI: Apnea–hypopnea index
APOC: Amsterdam Positional Obstructive Sleep Apnea Classification
ArTH: Respiratory arousal threshold
BMI: Body mass index
BSP: Best sleeping position
CC: Cartwright Classification
CI: Confidence interval
CPAP: Continuous positive airway pressure
CT90: Cumulative time of oxygen saturation < 90%
CVD: Cardiovascular disease
EDS: Excessive daytime sleepiness
ESS: Epworth Sleepiness Scale
HC: Hip circumference
HOMA-IR: Homeostasis model assessment of insulin resistance
LSaO2: Lowest oxygen saturation
MAI: Microarousal index
MS: Metabolic syndrome
NC: Neck circumference
NPOSA: Non-positional obstructive sleep apnea
NREM: Non-rapid eye movement sleep
ODI: Oxygen desaturation index
OR: Odds ratio
OSA: Obstructive sleep apnea
POSA: Positional obstructive sleep apnea
PSG: Polysomnography
REM: Rapid eye movement sleep
SaO2: Oxygen saturation
si-POSA: Supine-isolated positional obstructive sleep apnea
sp-POSA: Supine-predominant positional obstructive sleep apnea
SWS: Slow wave sleep
TST: Total sleep time
WC: Waist circumference
WSP: Worst sleeping position
