# Supplementary figures and images for: Prevalence, characteristics, and respiratory arousal threshold of positional obstructive sleep apnea in China: a large scale study from Shanghai Sleep Health Study cohort
Source: Respir Res. 2022 Sep 12;23:240. doi: 10.1186/s12931-022-02141-3 (PMC9465879; doi:10.1186/s12931-022-02141-3)

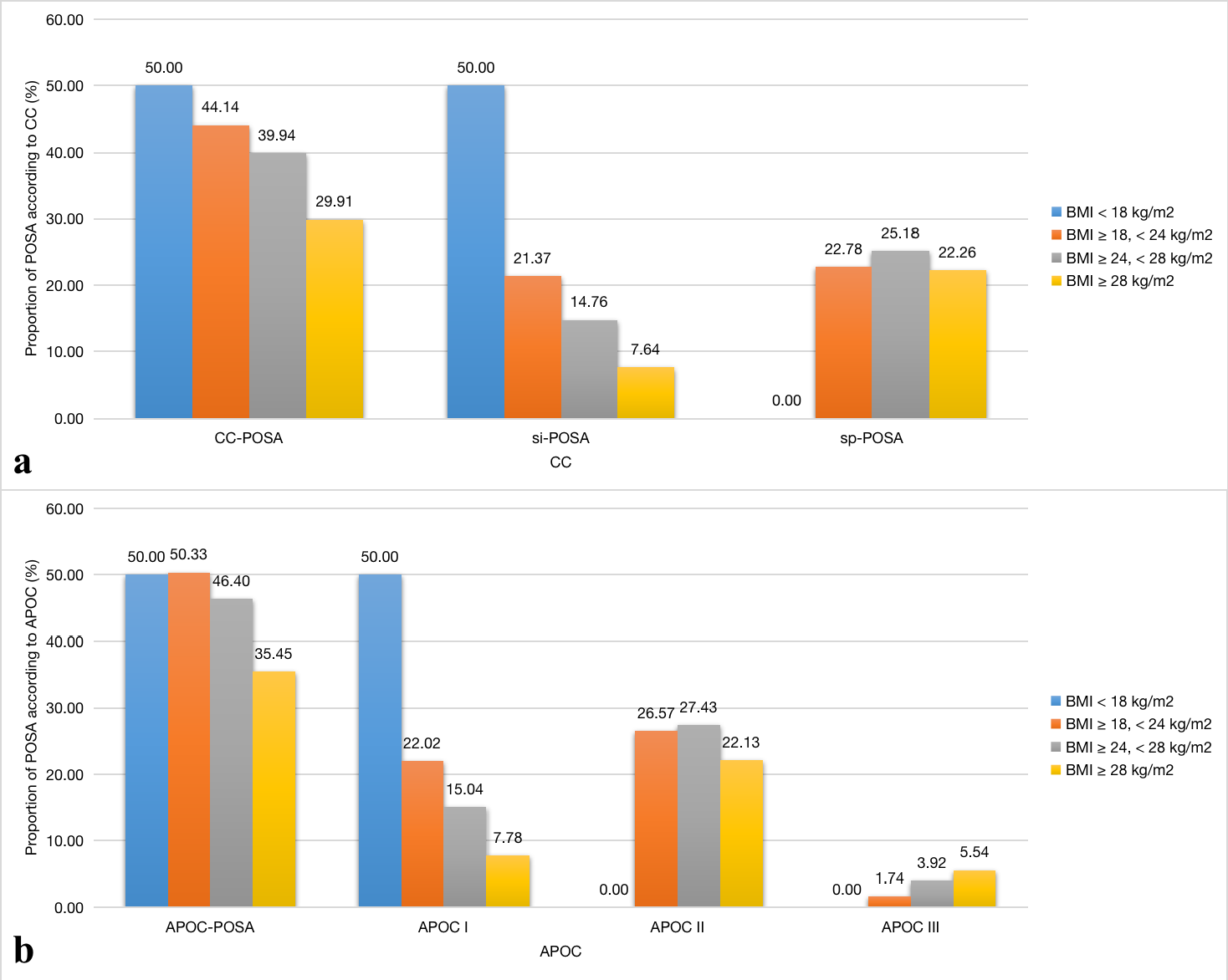

Supplement: Supplementary file 1 — Additional file 1: Figure S1. Prevalence of CC-POSA (a) and APOC-POSA (b) by BMI. APOC, Amsterdam Positional Obstructive Sleep Apnea Classification; BMI, body mass index; CC, Cartwright Classification; POSA, positional obstructive sleep apnea; si-POSA, supine-isolated positional obstructive sleep apnea; sp-POSA, supine-predominant positional obstructive sleep apnea. [file 12931_2022_2141_MOESM1_ESM.tif]
