# Supplementary material for: Prevalence, characteristics, and respiratory arousal threshold of positional obstructive sleep apnea in China: a large scale study from Shanghai Sleep Health Study cohort
Source: Respir Res. 2022 Sep 12;23:240. doi: 10.1186/s12931-022-02141-3 (PMC9465879; doi:10.1186/s12931-022-02141-3)
Supplement: Supplementary file 2 — Additional file 2: Table S1. Clinical and Sleep Characteristics of All Subjects (n = 7110). [file 12931_2022_2141_MOESM2_ESM.docx]

Additional file 2: Table S1. Clinical and Sleep Characteristics of All Subjects (n = 7110)

| ﻿Characteristics | NOSA (n = 1362) | OSA (n = 5748) |
| --- | --- | --- |
| Demographic and clinical characteristics |  |  |
| Men, n (%) | 823(60.43) | 4813(83.73)*** |
| Age, yrs | 37(30-48) | 43(35-54)*** |
| BMI, kg/m2 | 23.94(22.02-26.20) | 27.08(24.91-29.41)*** |
| NC, cm | 37(34-39) | 40(38-42)*** |
| WC, cm | 87(80-94) | 97(91-104)*** |
| HC, cm | 97(92-101) | 102(97.50-107)*** |
| WHR | 0.89(0.84-0.94) | 0.95(0.91-0.99)*** |
| SBP, mmHg | 120(118-121) | 121(120-135)*** |
| DBP, mmHg | 80(77-82) | 80(78-87)*** |
| Hypertension, n (%) | 140(10.28) | 1570(27.31) |
| Diabetes mellitus, n (%) | 80(5.87) | 485(8.44)*** |
| CVD, n (%) | 58(4.26) | 451(7.85)*** |
| MS, n (%) | 85(6.24) | 1017(17.69)*** |
| Hyperlipidemia, n (%) | 91(6.68) | 1000(17.40)*** |
| Smoking, n (%) | 286(21) | 1155(20.09)* |
| Alcohol consumption, n (%) | 525(38.55) | 3028(52.68)** |
| Snoring score, point | 4(1-6) | 7(5-9)*** |
| ESS, point | 4(0-9) | 8(3-13)*** |
| EDS, n (%) | 198(14.54) | 2033(35.37)*** |
| Biochemical indicators |  |  |
| Fasting glucose, mmol/L | 5.05(4.69-5.38) | 5.31(4.94-5.86)*** |
| Glucose 120 min, mmol/L | 8.82(6.12-13.44) | 8.07(6.28-11.56)*** |
| Fasting insulin, µU/mL | 7.71(5.37-11.31) | 11.85(7.93-17.54)*** |
| Insulin 120 min, µU/mL | 54.59(29.28-92.81) | 69.59(42.43-122.73)*** |
| HOMA-IR | 1.62(1-2.51) | 2.67(1.61-4.24)*** |
| TC, mmol/L | 4.37(3.80-4.99) | 4.77(4.19-5.40)*** |
| TG, mmol/L | 1.17(0.78-1.75) | 1.70(1.19-2.46)*** |
| HDL, mmol/L | 1.09(0.95-1.28) | 1.01(0.88-1.16)*** |
| LDL, mmol/L | 2.63(2.14-3.14) | 2.97(2.46-3.51)*** |
| ApoA-1, g/L | 1.08(0.96-1.23) | 1.05(0.94-1.18)*** |
| ApoB, g/L | 0.74(0.64-0.87) | 0.86(0.74-0.99)*** |
| ApoE, mg/dL | 3.86(3.20-4.76) | 4.34(3.54-5.48)*** |
| Lp(α), mg/dL | 8(4.10-17.10) | 7.40(3.87-15.60)*** |
| ApoA/ApoB | 1.46(1.19-1.81) | 1.23(1.04-1.47)*** |
| PSG |  |  |
| Mild OSA, n (%) | / | 1091(18.98) |
| Moderate OSA, n (%) | / | 1173(20.41) |
| Severe OSA, n (%) | / | 1671(29.07) |
| Extreme severe OSA, n (%) | / | 1813(31.54) |
| AHI, events/h | 1.80(0.60-3.30) | 38.90(18.73-60.20)*** |
| OAHI, events/h | 0.28(0-0.96) | 18.36(7.27-36.49)*** |
| Longest time of obstructive respiratory event, second | 16.85(0-27) | 53(37.50-66.50)*** |
| AHIREM , events/h | 1.30(0-5.20) | 45.20(20.70-61.20)*** |
| AHINREM, events/h | 1.60(0.60-3.10) | 38.20(17.20-60.50)**** |
| Supine AHI, events/h | 2.10(0.60-4.20) | 41.90(14-64.70)*** |
| Non-supine AHI, events/h | 0.86(0-2.10) | 26(9.30-54.08)*** |
| ODI, events/h | 2(0.80-3.70) | 38.40(18.50-61)*** |
| Mean SaO2, % | 96(95-97) | 94(92-95)*** |
| LSaO2, % | 92(90-95) | 78(68-84.50)*** |
| CT90, % TST | 0(0-0.04) | 5.97(1.40-19.54)*** |
| TST, min | 418(360.38-456.50) | 419.50(363-465)* |
| Supine Time, % TST | 89.12(46.86-92.61) | 74.85(41.72-84.40)*** |
| Sleep efficiency, % | 94.45(85.71-98.87) | 95.56(88.11-99.12)** |
| N1, % TST | 13.60(6.90-22.10) | 15.70(7.50-25.40)*** |
| N2, % TST | 49.70(36.10-58.50) | 51.10(37.80-60.70)*** |
| SWS, % TST | 14.40(5.83-22.30) | 11.40(4.20-19.80)*** |
| REM, % TST | 11.35(4.90-16.20) | 10(5.80-14.50)*** |
| MAI, events/h | 12.90(8.30-21.10) | 24.90(14-43)*** |
| Components of ArTH score |  |  |
| AHI <30 events/h, n (%) | / | 2264(39.39) |
| LSaO2> 82.5%, n (%) | / | 1880(32.71) |
| Proportion of hypopneas>58.3%, n (%) | / | 1400(24.36) |
| Absolute fraction of hypopnoeas, % | / | 31.76(11.03-57.89) |
| ArTH Score, cmH2O | / | -20.27(-27.86-[-13.88]) |
| Proportion with ArTH score ≥2, n (%) | / | 1803(31.37) |
| In male, n (%) | / | 1366/4813(28.38) |
| In female, n (%) | / | 437/935(46.74) |

Abbreviations: OSA, obstructive sleep apnea; NOSA, non-obstructive sleep apnea; BMI, body mass index; NC, neck circumference; WC, waist circumference; HC, hip circumference; WHR, waist hip ratio; SBP, systolic blood pressure; DBP, diastolic blood pressure; CVD, cardiovascular diseases; MS, metabolic syndrome; ESS, Epworth Sleepiness Scale; EDS, excessive daytime sleepiness; HOMA-IR, homeostasis model assessment of insulin resistance; TC, total cholesterol; TG, triglyceride; HDL, high-density lipoprotein cholesterol; LDL, low-density lipoprotein cholesterol; ApoA-I, apolipoprotein A-I; ApoB, apolipoprotein B; ApoE, apolipoprotein E; Lp(a), lipoprotein (a); PSG, polysomnography; AHI, apnea hypopnea index; OAHI, obstructive apnea hypopnea index; AHIREM, apnea hypopnea index in rapid eye movement stage; AHINREM, apnea hypopnea index in non-rapid eye movement stage; ODI, oxygen desaturation index; SaO2, oxygen saturation; LSaO2, lowest oxygen saturation; CT90, the cumulative time spent at oxygen saturation below 90% in total sleep time; TST, total sleep time; SWS, slow wave sleep; REM, rapid eye movement; MAI, micro-arousal index; ArTH, respiratory arousal threshold. * indicated p-value＜0.05 between OSA and NOSA. ** indicated p-value＜0.05 between OSA and NOSA. *** indicated p-value＜0.001 between OSA and NOSA.
